# General Medicine and Therapeutics

**Published:** 1896-02

**Authors:** 


### GENERAL MEDICINE AND THERAPEUTICS.

EDITED BY DR. LUTHER B. GRANDY.

### THE TOPOGRAPHY OF CONSUMPTION.

A review of the census returns of 1890 reveals some interesting and startling facts. The total nnmber of deaths reported from consumption was 102,199, a proportion of 121.49 per 1,000 of all deaths. The five years' record of the large cities gives Boston the largest percentage of mortality, 164.88 per 1,000 deaths. Chicago's proportion was 83.11 per 1,000.

So far as the States and Territories are concerned, New York returns the greatest number of deaths by consumption, 14,854; Indian Territory, the least, 22. If some of the States having the largest mortality were grouped consecutively, they would stand as follows: New York, Pennsylvania, Ohio, Massachusetts, Illinois, Tennessee, Missouri, Kentucky, Indiana, New Jersey, Virginia, California, Michigan, Maryland, North Carolina, Alabama, Georgia, South Carolina, Texas, and Wisconsin.

With the exception of California, the so-called climatic States present a good showing, Colorado standing thirty-fifth in the list, Florida thirty-seventh, Minnesota twenty-fourth, North Dakota forty-first, and South Dakota thirty-eighth. Of the New England States, Maine, New Hampshire, and Vermont are comparatively free from consumption, according to the mortality statistics.

The question arises, why is New York the storm center from which the current of consumption flows toward the Middle States? It cannot be due to climatic causes entirely, because the contiguous States have almost identical climatic conditions. On the other hand it is a well known fact that the more dense the population and the higher the degree of civilization, the more prevalent the scourge becomes. This fact is strikingly brought out by following the line of the progress of the disease in its course throughout the United States. If one traces the line through the States that have been mentioned he will see that the disease is concentrated at the centers of civilization. The social conditions that produce anxiety, mental strain, overcrowding, bad sanitation, debauchery, the struggle for existence, render the human kind less able to resist the onslaught of the consumptive germs. The States that are freest from consumption are those where the peeple live more out of doors, where the struggle for existence is less violent, and there is less need of improved sanitation.

The influence of heredity is another factor to be considered. The families that reside in the pest-ridden States are, in the majority of cases, the direct descendants of a tuberculous ancestry. Often the successive families occupy for generations the same dwellings that have harbored consumptives, and being susceptible to the disease they succumb to its influence.

A fact of interest to the climatologist is that the States that have the greatest amount of humidity do not seem to be over-ridden with consumption, as Washington and Michigan. The unfortunate States, however, are those of a low or medium altitude.

A glance at the Southern States emphasizes the fact that the racial predisposition of negroes to the disease increases the rate of mortality in the States where the colored population is proportionately great.

There are many other considerations in the study of the distribution of phthisis pulmonalis that emphasize the necessity of educating the public to the fact that the best way to stamp out the disease is to institute such hygienic and social measures as will improve the physical well-being of the race and thus enable it to better resist the invader.

Centuries before the discovery of the bacterial causation of the disease, Italy enforced sanitary laws that lessened the frightful mortality by consumption to a low degree.—*North American Practitioner*.

---

### TYPHOID FEVER IN YOUNG CHILDREN.

The occurrence of typhoid fever in young children has been a subject of discussion in several pediatric societies during the past two years. The evidence adduced renders it clear that the disease is extremely rare during the first two years of life, and uncommon under three years. Several gentlemen who have expressed the belief that it was not uncommon in "children" have evidently not been able to restrict themselves to children under three years. We are not aware that the claim has been made that it is particularly rare above that age. The records of four of the largest children's hospitals of the country disclosed the fact that not a single patient under three years of age had been admitted with typhoid fever for many years. Of these the New York Foundling Hospital furnished the most striking proof. Eleven hundred of the eighteen hundred children continuously under its care are "boarded out," in the city and surrounding towns, and are returned to the hospital when ill. Not a single case has been seen in this institution for twenty years, the facts being vouched for by Drs. Northrup, O'Dyer, ard J. Lewis Smith, and corroborated by 2,000 autopsies of Dr. Northrup.

The reasons thus far given to account for this insusceptibility are unsatisfactory. This is the age at which children are especially susceptible to intestinal diseases. Marked susceptibility to such diseases rapidly diminishes after two and a half years, the age at which typhoid susceptibility begins. If typhoid does not occur in infants because they are fed on sterilized milk and boiled water, why do they die from summer diarrhea in such appalling numbers? The germs of both diseases enter at the same portal—the digestive tract.

The condition which is most commonly mistaken for typhoid fever is, unquestionably, catarrhal pneumonia, a disease in which the physical signs are often obscure, and the rational symptoms indefinite. In young infants it is prone to pursue a prolonged course. Grippe, with slowly developing pneumonia, has also been mistaken for typhoid. The febrile conditions resulting from indigestion or simple gastro-intestinal disease may be the source of a similar error. It is certain also that pleurisy with effusion, and other obscure pulmonary and pleuritic disorders have been overlooked, and the fever accompanying them has been attributed to typhoid. The fact that a continued fever in a child may be due to tuberculosis should never be forgotten.

Typhoid fever is so rare in young children and is so closely simulated by other febrile conditions, that we are in full accord with Dr. Northrup's desire to encourage a healthy skepticism as to typhoid fever in an infant, in the absence of an epidemic, when the symptoms are not present which would lead to a diagnosis in an adult.—*Archives of Pediatrics.*

---

### GOITRE AND THE THYROID BODY AND EXTRACTS.

From a consideration of the history of this subject, and an analysis of the cases which have been presented thus far, the *British Medical Journal* thinks the following conclusions justifiable:

1. Thyroid products produce marked physiological effects upon the nervous and circulatory systems, as indicated by headache, dizziness, pains in other portions of the body, and great weakness, and by flushing of the face and rapidity of the heart's action.

2. Some of these unpleasant symptoms usually occur when a daily dose is reached corresponding to one and a half or two entire thyroid glands of the sheep.

3. If the administration of the remedy in doses that cause such symptoms is continued for a few days, constitutional effects are produced indicating that persistent use of doses from six to twelve grains of the dried thyroid (equivalent to one or two thyroid glands) three times daily might produce fatal results.

4. Desiccated thyroid glands appear quite as active as the liquid extracts, and more stable.

5. Internal administration appears quite as effective as hypodermic medication.

6. For internal use, the adult dose of the desiccated thyroids should not exceed two grains three times daily at first, but the dose may be gradually increased to two or three times this quantity, provided it does not cause unpleasant symptoms. There is no evidence that moderate doses have an injurious effect.

7. The remedy in some cases has a pronounced effect on the body weight, but this is very uncertain, and varies so greatly in different persons, and in the same individual at different times, that there is strong reason for suspecting that the loss of weight which sometimes follows this administration may be due entirely to disturbance of the digestive organs.

8. In the treatment of myxodema the remedy has undoubted value, and appears to benefit quite a large percentage. In these cases it is probable that the best results will be obtained by giving it at intervals for a long time.

9. In exophthalmic goitre the remedy causes rapid reduction in the size of the gland, but it has no perceptible effect upon the exophthalmia, and it apparently aggravates the heart symptoms. In this disease it must be used guardedly, and its effects must be carefully watched.

10. In many cases of goitre, internal administration of full doses of the products of the thyroid is followed by a most remarkable diminution in the size of the diseased gland. Improvement or cure may confidently be expected in seventy-six per cent. of the cases; but sufficient time has not yet elapsed to determine what the final result will be. It is probable that cystic growths in the thyroid gland would not be influenced by this remedy.

11. Clinical experience has not yet demonstrated that this remedy is of value in other diseases, but its effect in diminishing the size even of very firm and hard enlargement of the thyroid gland would certainly justify experimentation in other directions.—*Canada Medical Record.*

---

### THE VALUE OF FAMILY HISTORY AND PERSONAL CONDITION IN ESTIMATING LIABILITY TO CONSUMPTION.

Under this title, Dr. E. J. Marsh, Medical Director of the New York Mutual Life Insurance Company, makes an interesting report to the company. From a careful analysis of the records of the company for a great many years, he believes that the following conclusions are warranted:

1. That the history of consumption in any member of the immediate family increases the probability of its appearance in an applicant.

2. That consumption in a brother or sister is at least of equal importance as when it has occurred in a parent.

3. The persons who are under the standard or average of weight are much more liable to consumption than those above the standard. That the peculiarity of constitution which is indicated by the inability to take and assimilate a proper amount of nutriment indicates a susceptibility to phthisis, or at least is a reasonable suspicion of such predisposition.

4. That persons who exhibit a robust and well developed body have little susceptibility to consumption.

5. That the personal condition of weight and robustness has far more value than the family history in diminishing the liability to consumption; therefore,

6. The evidence presented by a well developed body may outweigh the suspicion attached to unfavorable family record.

7. That these influences of family history and personal weight are of the same grade for every age, and their importance is not lessened by the fact that the individual has reached middle life.

In deciding upon the eligibility of an applicant for life insurance, in whose case there is a suspicion of future danger from consumption, his personal condition is of the first, and his family record of the second importance. Whenever he presents a robust physical appearance, with a weight at least equal to the standard or average as given in our tables, he may be accepted, notwithstanding any taint in the record of his family. In our experience such persons have a small liability to consumption, although not protected from it. If, however, his weight does not come up to the average and he gives a history of consumption in brothers, sisters, or parents, he is to be regarded as an unfavorable risk. This does not mean that all such persons are to be absolutely excluded from insurance, but each case must be carefully scrutinized, and the decision based upon the circumstances of occupation, character, past history, etc. When these are favorable, insurance may be given by limiting the amount or modifying the form of policy; when unfavorable, the applicant should be either postponed until he has gained sufficient weight, or else be absolutely rejected.

---

### PHYSICIANS AND MALPRACTICE.

So many suits for malpractice or damages have lately been instituted against physicians in different parts of the country that the following points from the *General Practitioner* will be found timely and interesting:

1. A physician is guilty of malpractice when serious injury results on account of his gross ignorance or gross neglect.

2. A physician is guilty of criminal malpractice when he administers drugs or employs any surgical procedure in the attempt to commit any crime forbidden by statute.

3. A physician is guilty of criminal malpractice when he willfully or intentionally employs any medical or surgical procedure calculated to endanger the life or health of his patient, or when he willfully or intentionally neglects to adopt such medical or surgical means as may be necessary to insure the safety of his patient.

4. A physician is civilly responsible for any injury that may result to patient under his care directly traceable to his ignorance or his negligence.

5. A physician is expected by the law to exhibit in the treatment of all his cases an average amount of skill and care for the locality in which he resides and practices; further than this he is not responsible for results, in the absence of an express contract to cure.

6. A physician is not relieved of his responsibility to render skillful and proper treatment or reasonable care and attention by the fact that his services are gratuitous.

7. A physician is not obliged to undertake the treatment of any case against his will; but having once taken charge, he cannot withdraw without sufficient notice to allow his patient to procure other medical assistance.

8. A physician having brought suit and obtained judgment for services rendered, no action for malpractice can be thereafter brought against him on account of said services.

9. A physician is relieved of all responsibility for bad results in connection with the treatment of a case when there can be proved contributory negligence on the part of the patient.

---

### THE WOODBRIDGE METHOD OF "ABORTING" TYPHOID FEVER.

On the appearance of the first symptoms of typhoid the treatment is begun as follows:

(No. 1.)

| | | |
|---|---|---|
| ℞ | Podophyllum resin | 1-960 gr. |
| | Mercurous chloride | 1-16 " |
| | Guaiacol carbonate | 1-16 " |
| | Menthol | 1-16 " |
| | Eucalyptol | 1-10 " |

One capsule of the above formula should be given every fifteen minutes during the first twenty-four hours, and in larger doses during the second twenty-four hours, if found necessary, so that during this and the succeeding twenty-four hours there may be secured five or six full and free evacuations of the bowels during each of these periods. On the third or fourth day the following treatment should be begun:

(No. 2.)

| | | |
|---|---|---|
| ℞ | Podophyllum resin | 1-960 gr. |
| | Mercurous chloride | 1-16 " |
| | Guaiacol carbonate | 1-4 " |
| | Menthol | 1-16 " |
| | Thymol | 1-16 " |
| | Eucalyptol | 1-10 " |

One capsule to be given every hour or two.

No. 1, as well as No. 2, should be given very freely at first, gradually reducing the frequency of the dose, the object being to gradually reduce the number of movements of the bowels until the temperature has dropped to normal, when the movements should be only one or two a day.

Should symptoms of ptyalism manifest themselves, the treatment should be promptly discontinued for a day or two, sodium or potassium chlorate prescribed, returning as soon as possible to No. 1 or No. 2. About the fourth or fifth day the treatment with No. 3 should be begun:

(No. 3.)

| | | |
|---|---|---|
| ℞ | Guaiacol carbonate | 3 grs. |
| | Thymol | 1 gr. |
| | Menthol | 1-2 " |
| | Eucalyptol | 5 m. |

One capsule every three or four hours, alternating with No. 1 or No. 2.

During all the course of treatment the patient should wash down each capsule with large quantities of distilled or sterilized water, or in case it is indicated, some good laxative or diuretic mineral water is applicable to the case. It is claimed that if this treatment is begun early, nothing more will be required, and if carefully and intelligently carried out will rarely fail to abort typhoid fever.

During the last year or two no small amount of discussion has been had relative to this treatment. Many of the medical profession denounce it as a fraud and others uphold it as a valuable form of treatment.—*Columbus Medical Journal.*

---

### SYPHILIS AND LIFE INSURANCE.

The Mutual Life Insurance Company of New York has issued a pamphlet on this subject, prepared by Dr. E. J. Marsh, Medical Director. Dr. Marsh says that "syphilis should be looked upon as an impediment, but not as an absolute bar to life insurance. It is an impediment that might and ought to be cleared away by satisfactory explanation. There is a presumption of non-insurability, and the burden of proof for the removal of this presumption should rest upon the applicant.

"It has been stated that the vast majority of syphilitics never have any lesions offering danger to life, provided they have taken proper treatment, but that in a small minority of cases dangerous tertiary symptoms recur. The endeavor should be to select the good and reject the bad only. I think this might be accomplished by acting according to the following suggestions:

"1. No case with a history of any primary venereal sore should be accepted until six months shall have elapsed after its first appearance. If, however, in the absence of all constitutional treatment, no other symptom, such as glandular enlargement, eruptions, mucous patches, may have appeared by this time, the applicant might be acceptable. If he has undergone any constitutional treatment, a further postponement of six months after the termination of such treatment is necessary.

"2. No person with a history of syphilis is insurable until after a proper course of treatment and the lapse of at least six years from the date of infection.

"3. No person can be accepted who may have any history or evidence of tertiary manifestations.

"4. On the other hand, a person may be accepted who gives a history of constitutional syphilis, provided the original disease may not have been severe; that he shall have undergone a prolonged and satisfactory course of treatment, and a period of six years may have elapsed since the initial lesion, during the last two of which no relapses have appeared, and no tertiary symptoms at any time."

---

### CAFFEINE IN DISEASES OF THE RESPIRATORY ORGANS.

Dr. E. Markham Skerritt strongly recommends (*Practitioner*) the use of caffeine in some affections of the respiratory organs, and especially in spasmodic asthma. The drug acts in two ways: first, directly in producing relaxation of spasm, and secondly, indirectly by aiding the heart to overcome the obstruction in the pulmonary circulation. Dr. Skerritt does not think that the value of caffeine, in relieving the paroxysms of spasmodic asthma, is at all generally realized.

He has found it successful when drug after drug had been tried in vain. He prescribes the citrate of caffeine in the dose, for an adult, of five grains, taken in a *cachet* or in water. He thinks the effervescing hydrobromate of caffeine inconvenient, and has never administered caffeine hypodermically. In acute bronchitis, caffeine will often relieve the dyspnea, and also in chronic bronchitis and emphysema. As the drug is a cardiac tonic, acting on the medulla and heart centers, it is of great service in acute respiratory troubles with threatening heart-failure. Some ill effects have been described by Lehmann, Pratt, and Routh, after doses varying from eight grains to one drachm; but the author has never witnessed any untoward results. Occasionally a condition of wakefulness is induced, but it is described as pleasant, the patient feeling wide awake, mentally active, and free from any disagreeable sensations. As a rule, patients sleep without difficulty after their nightly dose of five or ten grains, and no evil effect has been noted in cases where the administration of the drug has extended over long periods—sometimes even years.—*Medical Chronicle.*

### ACUTE LOBAR PNEUMONIA.

In an entertaining article on this subject, *American News and Practitioner,* Dr. John A. Lewis offers the following conclusions :

1. The disease is an infectious fever and has a definite course to run.

2. Its course is a short one, usually ending in an abrupt crisis about the seventh day.

3. Our plain duty is to keep life in the patient until nature effects the cure.

4. No routine plan of treatment can be adopted suited to every case.

5. The majority of deaths result from heart exhaustion.

6. Rapid action of the heart together with the increased power required to overcome the resistance in the pulmonary circulation are the two main factors producing heart exhaustion.

7. The fever is the result of the growth of bacteria, attended by the formation of poisonous chemical products in the system.

8. As yet we have no safe or certain agent by which we can destroy the diplococcus pneumoniæ in the human body.

9. An overdistended right ventricle from pulmonary obstruction is one of the greatest sources of danger.

10. Pain, cough, sleeplessness, are extremely wearing upon the vital forces of the patient.—*Western Med. Reporter.*

---

### A GOOD GENERAL TONIC.

| | | |
|---|---|---|
| ℞ | Liq. ferri citratis | oz. j. |
| | Tinct. gentianæ comp | |
| | Tinct. cinchonæ comp | aa oz. iss. |
| | Strych. sulph | gr. j. |
| | Pepsin | dr. iss. |
| | Syr. acidi hydriodici | oz. iijss. |

M. Sig.—Take a teaspoonful after each meal, in wineglass of water.—*World.*

### HOW THE ORIFICIAL SURGEONS CURE A COUGH.

A late issue of the *Journal of Orificial Surgery*, it appears, contained an article of a Dr. Cooper, of Kansas, entitled, "A Cough Cured." Commenting upon the case, the *Northwestern Lancet* says :

"How a cough is cured in Kansas may be of interest to all, even if the cure be not imitated. The writer of the paper, while passing his vacation at Manitou, was consulted by a vigorous, blue-eyed, light-haired Swedish girl of twenty-six summers, on account of a cough and various nervous disturbances.

"The cure was as follows : First of all the lady must reside in the same house with the physician. Then came phosphate of iron, chloride of potash, galvanization of the body, faradization and dilatation (!) of the rectum. There followed galvanization of the solar plexus and of both vagi, nasal sprays, verbascum oil in the ears, and finally excision of the hymen and further dilatations and faradizations as already described, together with regulation of the diet and inhalations of oxygen, and the cure burst in upon this treatment like an avalanche in the mountains of the moon or an earthquake in the Sierra Nevadas. A poem by an unnamed writer closes this medical history."

### LOTION FOR CHAPPED HANDS.

| | | |
|---|---|---|
| ℞ | Glycerin | 8 fluidounces. |
| | Cologne | 2 fluidounces. |
| | Borax | 2 ounces |
| | Alcohol | 2 fluidounces. |
| | Camphor-water | 16 fluidounces. |

Mix, and color with tincture cudbear to suit.

For preparations having an alkaline reaction, solution of carmine can be used to color. For preparations containing acids, use tincture of cudbear, cosine, or anilin red.—*Bulletin of Pharmacy.*

### KEEP YOUR MOUTH SHUT.

The *Family Doctor* says that this is the secret of avoiding colds. The man or woman who comes out of an overheated room, especially late at night, and breathes through the mouth, will either catch a bad cold or irritate the lungs sufficiently to cause annoyance and unpleasantness. If people would just keep their mouths shut and breathe through their noses, this difficulty and danger would be avoided. Chills are often the result of people talking freely while out of doors just after leaving a room full of hot air, and theater-goers who discuss and laugh over the play on their way home are inviting illness. It is, in fact, during youth that the greater number of mankind contract habits or inflammation which make their whole life a tissue of disorders.

